# Endothelial defects unveil cardiovascular phenotype in iPSC-based disease modelling across three generations of a DiGeorge syndrome family

**DOI:** 10.21203/rs.3.rs-7141852/v1

**Published:** 2025-07-18

**Authors:** Tünde Berecz, Brigitta Szabó, Ábel Fóthi, Andrea Ágnes Molnár, Kristóf Árvai, Leila Holub, Irén Haltrich, Katalin Vincze, György Várady, Tamás I. Orbán, János Kriston-Vizi, Béla Merkely, Laura Kerosuo, János M. Réthelyi, Gábor Földes, Ágota Apáti

**Affiliations:** Institute of Molecular Life Sciences, HUN-REN RCNS, Budapest, Hungary; Institute of Molecular Life Sciences, HUN-REN RCNS, Budapest, Hungary; Institute of Molecular Life Sciences, HUN-REN RCNS, Budapest, Hungary; Heart and Vascular Center, Semmelweis University, Budapest, Hungary; Department of Pathology and Experimental Cancer Research, Semmelweis University, Budapest, Hungary; Department of Pathology and Experimental Cancer Research, Semmelweis University, Budapest, Hungary; 2nd Department of Pediatrics, Semmelweis University, Budapest, Hungary; Institute of Molecular Life Sciences, HUN-REN RCNS, Budapest, Hungary; Institute of Molecular Life Sciences, HUN-REN RCNS, Budapest, Hungary; Institute of Molecular Life Sciences, HUN-REN RCNS, Budapest, Hungary; Laboratory for Molecular Cell Biology, Medical Research Council, University College London; Heart and Vascular Center, Semmelweis University, Budapest, Hungary; National Institute of Dental and Craniofacial Research, National Institutes of Health, Bethesda, MD, USA; Department of Psychiatry and Psychotherapy, Semmelweis University, Budapest, Hungary; Heart and Vascular Center, Semmelweis University, Budapest, Hungary; Institute of Molecular Life Sciences, HUN-REN RCNS, Budapest, Hungary

**Keywords:** DiGeorge syndrome, pluripotent stem cells, cardiovascular disease models

## Abstract

DiGeorge syndrome (DGS) is caused by a microdeletion on chromosome 22, leading to variable disease phenotypes and severity and often involving congenital cardiovascular disease. In this work, we performed a detailed study of DGS patients with increasing severity within a family of three generations. Genetic analyses revealed no correlation between DGS severity and the size of the deletion or the number of overall genetic variants; however, we identified potentially high-impact variants, indicating a role for these genes in predisposition to the disease. For a phenotypic analysis, induced pluripotent stem cells (iPSCs) were generated and differentiated into functional cardiomyocytes and endothelial cells using blood cells from family members. In DGS patients, the iPSC-derived cardiomyocyte clusters during differentiation showed morphological differences in sarcomere arrangement along with lower cardiac connexin-43 expression. These findings indicate an impaired structural development due to an altered expression of gap junction proteins and cardiomyocyte-matrix connections as a basis of cardiovascular pathology. In endothelial cells differentiated from iPSCs of the DGS patients, we found an aberrant vascular phenotype. Vascular defects were observed in differentiation and migration, as well as in endothelial tubular morphology, accompanied by altered transcription patterns for angiogenesis and vascular integrity pathways. These results suggest that DGS-specific cell types differentiated from hiPSC show major alterations and may be used to uncover genotype-phenotype correlations with the potential to reveal the molecular basis of the clinical manifestations in DGS.

## 1. INTRODUCTION

DiGeorge syndrome (DGS), also known as 22q11.2 deletion syndrome (22q11.2DS) or velocardiofacial syndrome, represents the most prevalent microdeletion syndrome, affecting 1 in 4000 live births. Prenatal invasive testing reveals that its occurrence could be as high as 1 in 1000 foetuses [1]. The syndrome arises from a monoallelic microdeletion on chromosome 22, predominantly due to non-allelic homologous recombination during meiosis. Notably, 90%-95% of these deletions occur *de novo*, indicating a significant rate of spontaneous deletion. Conversely, 5–10% of cases inherit the disease in an autosomal dominant fashion [2]. The typical 1.5-3 Mb microdeletion on chromosome 22's long arm leads to haploinsufficiency, manifesting in a wide array of phenotypic outcomes. Key symptoms include immunodeficiency due to absent or aplastic thymus, hypocalcaemia caused by hypoplasia or absence of the parathyroid gland and neuropsychiatric disorders (ADHD in childhood and schizophrenia in adults).

In a large number of cases (64%) congenital heart disease also affects the DGS patients [3]. The most frequent cardiac defects are interrupted aortic arch, truncus arteriosus, Tetralogy of Fallot (ToF), ventricular septal defects and vascular rings. The affected organ systems vary among patients, with symptoms ranging broadly from mild to lethal clinical manifestations. Despite the variability in clinical presentation, the severity of symptoms does not correlate with the deletion's size but tends to increase with inheritance [4]. The 3 Mb long 22q11.2 region encompasses 45 protein-coding genes, seven miRNAs, and ten non-coding genes, along with additional predicted coding and non-coding genes [5]. Proposed mechanisms underlying the heterogeneous clinical manifestations with the same size of 22q11.2 deletion are gene dosage effects, gene variants in the intact allele within the 22q11.2 region, other gene variants outside the 22q11.2 region, and epigenetic and environmental factors.

Human pluripotent stem cells (hPSC) and their derivatives provide promising opportunities for studying disease-related phenotypes *in vitro*. These approaches are particularly important when human cell types cannot be investigated directly in the long term, or no appropriate animal models are available [6]. Here, in addition to a detailed clinical and genetic characterization of the patients and their healthy relatives from the family, we established an iPSC-based model of 22q11.2 deletion disease for DGS using cells from three patients and two healthy relatives. As a disease-specific model, we focused on the generation of cardiovascular cells from the iPSC and carried out a comparative investigation of genetics and phenotypes from diseased and healthy cell derivatives.

## 2. RESULTS

### 2.1. Clinical description of a three-generation family with DGS.

**Figure 1:**
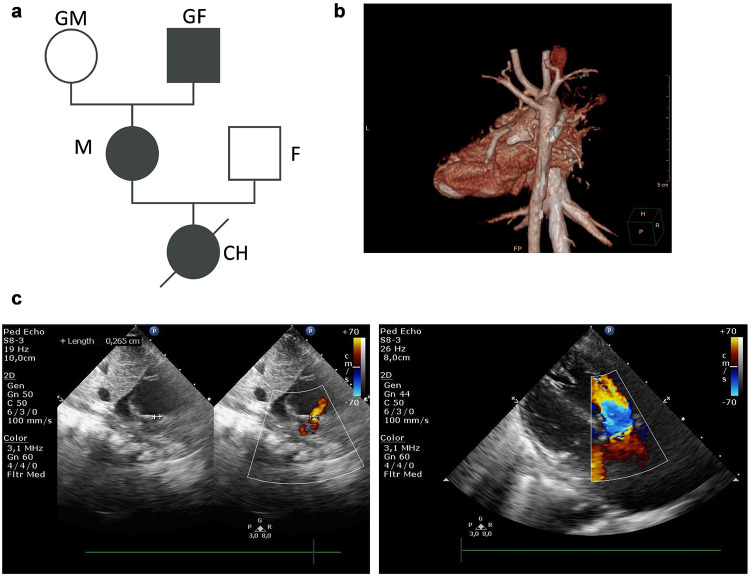
Characterisation of the DiGeorge family. **a.**Pedigree of the family involved in the study. Clinical symptoms of patients show increasing severity when the disease is inherited (see also Table 1). **b-c**. Cardiac anomalies assessed by echocardiography, invasive angiography and thoracic CT scan. Findings from imaging in child was pulmonary artery atresia with missing pulmonary trunk and bilateral complete disruption of main pulmonary artery; presence of branch pulmonary artery stenosis, lack of left pulmonary artery, number of bronchopulmonary segments supplied by native left pulmonary arteries and the distribution of major aortopulmonary collateral arteries (MAPCAs) originating from artery anonima; narrow right side MAPCA originating from descending thoracic aorta; and right aortic arch with aberrant retroesophageal left artery with subclavian artery, without Kommerell’s diverticulum. A haemodynamic study performed on the left MAPCA demonstrated significant stenosis in the distal segment of the left MAPCA, which underwent palliative dilatation and stent implantation (a 4/15mm and a 5/20mm sinus-Superflex self-expanding stent), and stenosis was also demonstrated at the origin of all three right gracilise MAPCAs. Type I atrial septal defect with a large left-right shunt; 3-4mm outlet type ventricular septal defect with a bidirectional shunt; 6-7mm (over which the aorta rides) were also shown. As pulmonary circulation was not ductus Botall-dependent, Prostin medication was suspended from day 3 after birth.

As inherited and transgenerational autosomal dominant recurrence of DGS is relatively low (~10% of patients), families with three generations are extremely rare. Indeed, only two families have been reported to date [7, 8], and no hiPSC lines were established from these. We screened the Hungarian registry of DGS patients and selected a family for further investigation. A family with three DGS patients from three generations (grandfather (GF), mother (M) and child (CH)) manifesting the disorder with different severity, and two healthy relatives (grandmother (GM) and father (F)) were enrolled in this study (Fig. 1a and Table 1). Mild clinical manifestation of GF consists of articulation disorder and minimal facial dysmorphia. He was treated with beta blocker for hypertension. Moderate presentation in M includes vascular ring (surgically corrected in childhood), hypocalcaemia and minimal facial dysmorphia. Clinical features of CH were severe and led to death at the age of 5 months (Table S1); she was diagnosed with ToF (ventricular septal defect, pulmonary atresia, right ventricular hypertrophy, misplaced aorta- Fig. 1b,c), atrial septal defect, asymmetrical brain ventricles, hypocalcaemia, hypoparathyroidism, and minimal facial dysmorphia (Table 1). Genetic FISH analysis diagnosed all three patients with DGS, but no additional, comprehensive genetic tests had been performed prior to this study. Of note, karyotype testing of PBMCs revealed a chromosomal translocation between chromosomes 6 and 12 in the father. However, when the breakpoints of such translocations do not occur within gene or regulatory element sequences, these typically do not result in phenotypic changes [9]. The father was healthy, so no further characterization of the breakpoint details was conducted, and the father was considered a healthy control. CH did not inherit the translocation.

### 2.2. Genetic variants in the family members and their potential impact on DGS severity.

**Figure 2:**
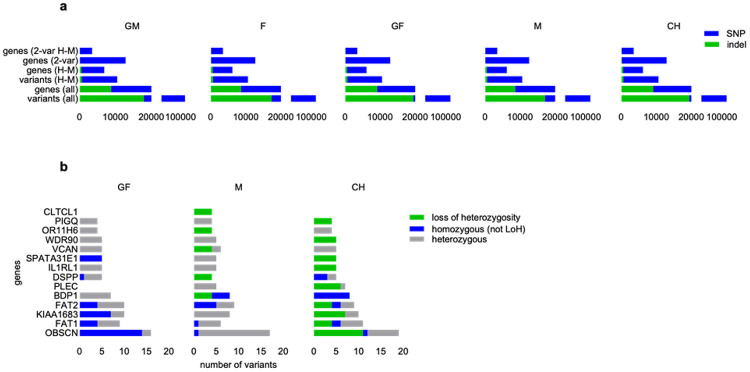
Analysis of whole exome sequencing data. **a.** For each family member, the columns (from bottom to top) show the number of all genetic variations, the number of all affected genes, the number of variations with High or Moderate impact and the number of affected genes, and finally the number of genes with more than 2 variations and more than 2 high or moderate impact variations compared to the human genome. **b**. High- or moderate impact genes that carry 4 or more homozygous variants due to Loss of Heterozygosity (LOH) in the mother or child. In the case of GF, LOH cannot be determined because parental data are not available.

Genetic variants within the exomes of family members may contribute to the variability in clinical symptoms observed in DGS, even among those with similar or identical 22q11.2 deletions. To explore the potential link between increasing disease severity across generations of patients with DGS, we conducted whole exome sequencing (WES) on hiPSC lines generated from the blood samples from family members. There were only minimal differences in the number of genetic variants, single nucleotide polymorphisms (SNPs) and insertions/deletions (indels), even when focusing solely on high- and moderate-impact variations (Fig. 2a). This suggests that the more severe disease phenotype in subsequent generations is not directly correlated with an accumulation of genetic alterations in the exome. We also investigated whether other genetic alterations, particularly variants in the remaining 22q11.2 region on the intact allele, contribute to the disease manifestations. While WES identified variations (SNPs and indels) within the 3 Mb DG region in all family members, we did not detect any high impact, de novo variants in the intact allele in the DG region in the cases of M and CH. However, we found high-impact variants in the DG region of more than one family member in the Claudin 5 (CLDN5), catechol-O-methyltransferase (COMT), Clathrin Heavy Chain Like 1 (CLTCL1), Scavenger Receptor Class F Member 2 (SCARF2), and T-Box Transcription Factor 1 (TBX1) genes (Table S3a). Of note, the CLDN5 variant was only homozygous in M (inherited from GM, who is heterozygous for this variant). For TBX1, only GF and CH carried the same C-T variant in a homozygous form, indicating that variations in certain genes may be more significant in the development of symptoms (Table S2a,b). We also assessed high impact *de novo* or loss of heterozygosity (LOH) mutations in DGS patients with cardiovascular symptoms (CH, Table S2b and M, Table S2c). Outside the DG region, we did not identify any variants that have been previously associated with DGS [https://pathcards.genecards.org/]. To further investigate the differences between M and CH, we examined the genes affected by high- or moderate-impact variants and their distribution (Fig. 2b), focusing on variants exhibiting four or more homozygous alterations. In CH, we identified a total of 51 such variations across nine genes, while M exhibited 20 variations distributed among five genes. For GF, we also identified these genes and several variants; however, these were less frequent and primarily presented in a heterozygous state. Thus, although no differences were found in the total number of genetic variations (Fig. 2a), the increased severity of the symptoms may be partially reflected in the accumulation of deleterious variations within specific genes.

Based on medical records and assessment, we tested selected genes that were in the disease similarity network of the 2575 diseases from the Human Phenotype Ontology (HPO) dataset based on their Mantis-ML 2.0 rankings across all genes. After hierarchical clustering, all features ranked by their clinical importance for the DGS cases (GF, M, CH) are shown as a heat map (Fig. S1). Scores of candidate genes, like those with loss-of-heterozygosity mutations identified by whole exome sequencing (WES), were presented with high gene scores (OBSCN 0.82; FAT1, 0.87; PLEC, 0.88; VCAN, 0.9 and TBX1, 0.96), strongly indicating a significant probability of these associated gene being causative for the DGS patients' cardiac phenotype trained across all balanced clinical datasets.

### 2.3. Generation and characterization of hiPSC lines from DGS patients.

Blood mononuclear cells were isolated from patients (GF, M, CH, all diagnosed with DGS) as well as healthy relatives (GM and F) and were reprogrammed into induced pluripotent stem cell (hiPSC) lines by a commercially available Sendai virus kit (Fig. S2a). Two CH clones and one clone from the other relatives were selected for further characterization. Karyotype testing revealed no chromosomal alterations in the hiPSC lines, except for the father, where a translocation between chromosomes 6 and 12 was also detected in the hiPSC (Fig. S2b). The established hiPSC lines expressed SSEA4 cell surface and OCT4 transcription factor pluripotency markers (Fig. 3a,b). Moreover, hiPSC colonies were positive for NANOG (Fig. S2c) and differentiated into all three germ layers, as demonstrated by immunohistochemistry (Fig. S2c) and realtime PCR (Fig. S2d). The genetic signature identity of hiPSC and hiPSC-matched blood samples was confirmed by STR analysis (Table S3)[10]. We proved sustained microdeletions in patient-derived hiPSC lines using MLPA. We found the same microdeletion in all DGS-derived samples (Fig. 3c). Human iPSC of DGS patients showed significantly lower mRNA expression of DGCR8 than those in controls (P = 0.005, Fig. 3e), consistent with the genetic background. In contrast, OCT4 expression did not vary between the cell lines (Fig. 3d).

### 2.4. Characterization of DGS family-specific hiPSC-derived cardiomyocytes.

Cardiac symptoms are the predominant manifestation in DGS. Thus, adapting a previously described protocol [11], cardiomyocytes were differentiated from hiPSC derived from five members of the DGS family (each family member was represented by a single clone, except for CH, for which two clones were evaluated) (Fig. 4a). Spontaneous beating of hiPSC-CM started between days 7 and 15 of differentiation. M and CH hiPSC-CM cultures showed different morphologies at day 10, such as markedly lower adhesion to the cell culture matrix surface and spherical or fibre-like morphology in the case of CH (Fig. 4b). Furthermore, the CH beating activity was also significantly the highest at that time point (Fig. 4c). Cardiac gap junction connexins Cx40 (GJA5) and Cx43 (GJA1) have been implicated to have a role in developing arrhythmia and cardiac anomalies, including ToF [12-14]. We observed that GJA1 mRNA levels were significantly lower in CH hiPSC-CM, suggesting an alteration in cell-cell-adhesion capacity, like those in clinical manifestation. No difference was observed in the expression of GJA5 (Fig. 4d). Metabolic selection and replating hiPSC-CM eliminated the morphological differences as they developed into stable monolayers of cell sheets with high purity (> 95% cardiac-specific TNNI3^+^ hiPSC-CM) at day 36 after the start of differentiation (Fig. 4e). The efficacy of the cardiac differentiation was confirmed by abundant mRNA expression for multiple cardiac markers: NKX2.5, TNNI3 and TNNI2, as well as subtype-specific markers such as MYL2 (ventricular), MYL7 (atrial) and HCN4 (pacemaker) and other cardiac markers such as GATA4, TBX5, NPPA, NPPB, CACNA1-H, RYR2, PLN and ISL1. An increase in mRNA levels of these markers, as well as a decrease in OCT4 compared to hiPSC, were comparable between family members (Fig. 4f). Spontaneous intracellular Ca^2+^ transients were assessed in the replated monolayer of hiPSC-CM using Fluo-4 Ca^2+^-sensitive dye on day 36. hiPSC-CM from DGS patients (GF and CH) showed a higher beating rate and frequency-dependent, hence shorter times for most calcium kinetics parameters, as compared with M and control GM and F (Fig. 4g-j). To characterize the structural properties of single hiPSC-CMs, we performed a high-content confocal image analysis based on TNTI3 immunocytochemistry (Fig. S3). All cells displayed structural features of the immature phenotype in terms of shape and sarcomeric organization. The morphological differences in sarcomere arrangement (entropy) and varying Ca^2+^-kinetics do not indicate that cardiomyocyte-autonomous alterations cause cardiac symptoms in DGS, suggesting that cell-cell and/or cardiomyocyte-matrix connections may play a more significant role in cardiac pathology.

### 2.5. Characterization of DGS family-specific hiPSC-endothelial cells (EC).

Endothelial cell changes may contribute to the clinical cardiovascular manifestations associated with DGS. Thus, we generated endothelial cells from each hiPSC line using Activin-A, BMP4, bFGF and VEGF_165_. CD31^+^ cells were isolated by FACS at day 12 and maintained until day 19 of differentiation (Fig. 5a). Based on our previous findings, hiPSC-ECs present high proliferative potential, self-repopulating activity, and *in vivo* vessel-forming ability. On day 12 of endothelial differentiation, M and CH hiPSC-EC formed thicker and less branched microvessels, as expressed by tube thickness and node area (Fig. 5b,c). Thus, this complex, aberrant *in vitro* vascular phenotype and related defects in endothelial cell differentiation and migration were dominant in hiPSC-EC from DGS patients with vascular malformations but not in the asymptomatic DGS individual or healthy members of the family (Table 1 and S1). Total tubular length and area of the formed endothelial microtubules (in proportion to the number of hiPSC-EC) were comparable in each hiPSC line (Fig. 5c). The observed significant differences in adhesion and cell-cell connectivity between endothelial cells *in vitro* in M and CH further support the differences highlighted by mRNA sequencing data (see below).

To gain a comprehensive understanding of cell-specific gene expression, an assessment of transcriptomics is also required. Bulk RNA sequencing was performed on undifferentiated hiPSCs (day 0), on day 5 of differentiation, and on sorted CD31 + hiPSC-ECs on day 19 from each hiPSC line. Principal component analysis (PCA) revealed a complete and comparable high-fidelity differentiation pattern of hiPSCs toward mesodermal (by day 5) and endothelial lineages (by day 19) in each line (Fig. 6a, rightmost panel). PCA further showed that in undifferentiated hiPSC cultures, the biological and technical replicates cluster closely together for all family members (note two CH clones exhibiting a tight clustering). This close association is not observed in the samples collected at day 5 any longer, suggesting that mesodermal commitment may be more heterogeneous than that in pluripotent or endothelial differentiation phases. By day 19, a notable separation is observed in the first principal component (PCA1) axis between DGS patients with vascular symptoms (M and CH) and their asymptomatic family members. A heat map indicates that the expression levels of genes within the 22q11.2 region were consistently lower in all three DGS patients compared to healthy controls at each time point during differentiation (Fig. 6b).

Bulk RNA sequencing analysis between healthy participants and patients with DGS revealed 2,200 upregulated and 1,500 downregulated genes, with a notable overrepresentation of upregulated genes (Fig. 6c). The most under-expressed gene was GSTM1 (9-fold, p = 10e^− 50^), whose reduction or complete loss is associated with the upregulation of intracellular and vascular cell adhesion molecules, such as VCAM-1 and ICAM-1 [15]. Ingenuity Pathway Analysis (IPA) on differentially expressed genes revealed that the expression patterns in hiPSC-EC *in vitro* reflect a clinical worsening of the disease between generations (Fig. 6d). By using activation z-scores, we identified distinct categories where given biological functions were affected in all three DGS patients (Fig. 6d diGeorge syndrome panel), patients with cardiovascular symptoms (Fig. 6d symptomatic diGeorge syndrome panel) or in patient (CH) with the most severe cardiovascular symptoms only (Fig. 6d lethal form of diGeorge syndrome panel). Functional and disease-associated terms related to angiogenesis and vasculogenesis, such as peripheral vascular disease and vascular lesion, were activated in all DGS patients, with a higher probability in M and CH. Several endothelial terms, such as endothelial development and endothelial migration, were activated in the cells of DGS patients with vascular symptoms, with higher activation scores in CH. Due to the role of endothelial cells in cardiac development [16], dysfunction of endothelial cells may contribute to abnormal heart development observed in CH [17].

These results demonstrate the accumulation of activated cardiovascular function, abnormal morphology, and disease-associated terms in endothelial cells of patients, in accordance with the observed increase in clinical symptoms throughout generations. Enriched Gene Ontology (GO) terms highlight the 15 GO processes with the highest gene ratios based on differentially expressed genes between healthy participants and patients with DGS (Fig. 6e). Notably, among the GO terms in CH and M, the three strongest positive deviations from control expression levels were related to cell-cell adhesion and connectivity. In the case of GF, who has no cardiovascular symptoms, immunological terms dominated. String-DB analysis showed an increasing clustering of the cytokine-chemokine system in M and, notably, in CH, compared to those in GF (Fig. 6f). Of note, chemokines are key not only for activating host immune responses but also for morphogenesis and wound healing [18]. The tissue reorganization-related GO hub noted in M and CH showed extracellular matrix arrangements, primarily affecting collagens, such as COL1A1. These findings underscore the significant alterations in gene expression and cellular phenotypes associated with DGS, particularly in relation to vascular symptoms and endothelial cell behaviour.

## 3. DISCUSSION

The clinical manifestations of 22q11.2 deletion syndrome encompass a wide spectrum, with over 180 identified phenotypic expressions contributing to its causal complexity. Notably, the variability in cardiac anomalies, including differences in severity and familial heterogeneity among individuals with identical deletions, underscores the challenges in clinical diagnosis and management. The aetiology of this phenotypic diversity is thought to involve stochastic processes, genetic variations within the remaining 22q11.2 chromosomal region, and additional genetic modifiers located elsewhere. The genetic architecture of the DiGeorge sequence further complicates modelling efforts in murine systems due to divergent genetic organization and the absence of human orthologous genes, limiting the translational capacity of these models [19].

In light of these complexities, hiPSC-based disease modelling emerges as a promising avenue for elucidating the underlying mechanisms of DGS. Initial hiPSC models primarily explored the neurological dimensions of the syndrome [20-22], with only a singular study delving into its cardiac implications, revealing the dose-dependent regulation of MEF2C expression by TBX1 [23]. Our research presents a novel patient-derived hiPSC model that accurately reflects the specific cardiovascular manifestations of DGS across three generations of a single family, all of whom bear the same 22q11.2 deletion yet display variable severity. This model prompts us to investigate a) the feasibility of studying DGS's cardiovascular aspects using a hiPSC-based approach; b) the potential to identify cellular phenotypes within hiPSC-derived cardiomyocytes and endothelial cells; and c) the capability to track the generational progression of disease symptoms.

Our experiments show that hiPSC, cardiomyocytes and endothelial cells can be generated from DGS of varying severity. The morphology of differentiating hiPSC-CM cultures was transiently different between DGS with cardiovascular clinical presentation (M and CH) and healthy cultures. This suggests that cardiomyocyte morphogenesis is altered during early differentiation in DGS patients with cardiovascular symptoms compared to asymptomatic family members. In early development, gap junction proteins like Cx43 (GJA1) are expressed in ventricles and the interventricular septum [14, 24]; we found that its molecular expression patterns were inhibited in differentiating CH hiPSC-CM. Disturbances of Cx43 levels have been shown to contribute to ToF [14, 25], which is in line with the set of clinical symptoms diagnosed only in the child. We have previously developed an optical flow-based method to quantify the dynamic behaviour of hiPSC-CM [26], and this method was applied to cardiomyocytes differentiated from members of the studied family at the early stage of differentiation (20 days); quantitative characterization of contractile cycles revealed no significant differences between CH hiPSC-CM and those from other family members [27]. However, the contraction speed and strain rate in M, as well as the contraction and relaxation phases and rest periods between cycles in CH, were altered. In more differentiated, pure hiPSC-CM cultures (at day 36), there were no significant differences in marker expressions or function (spontaneous beating and Ca^2+^-transients were found in every case), implicating that DGS-related cardiac malformations, such as ToF, may originate from structural abnormalities involving cell-cell and cell-ECM connections in the heart rather than distinct cardiomyocyte function. Other studies also report normal myofilament function in patients with ToF [28], but multiple extracellular matrix components are differentially expressed in human foetal hearts with ToF [29]. Of note, GF and CH hiPSC-CM had a faster spontaneous beating than those from other family members. This phenotype was not associated with any cardiovascular developmental disease, which may reflect the limitations of direct head-to-head comparisons of clinical symptoms, such as multifactorial heart rate regulation and in vitro phenotypes in our settings.

Controlled endothelial adhesion is vital for angiogenesis and maintaining vascular integrity, and it involves adhesion molecules that mediate cell-cell and cell-ECM interactions. Disruptions in this process can contribute to the development of vascular malformations. However, the direct link between endothelial adhesion and vascular anomalies associated with DGS, particularly severe congenital heart defects (CH) and symptoms such as vascular rings (M), has not been thoroughly documented. Immune deficiencies characterize DGS due to thymic hypoplasia or aplasia, which is clinically evident in M and CH. Given the role of the endothelium in immune cell trafficking, alterations in endothelial adhesion may also impact immune cell migration and function, potentially contributing to immune deficiencies. Structural and transcriptomics profiles of *in vitro* hiPSC-EC cultures were well associated with the clinical manifestation of DGS for each family member. The migration and adhesion capacity of hiPSC-EC was progressively attenuated in the three affected generations, resulting in a disorganized vascular network with enlarged nodes and branches in M and CH.

mRNA sequencing analysis of both undifferentiated hiPSCs and differentiated hiPSC-ECs at multiple stages elucidated the underlying expression patterns during differentiation. A combination of whole-exome sequencing data from hiPSC lines and RNA sequencing data from hiPSC-EC was used to assess the contribution of copy number variations and genetic variants, in addition to the 22q11.2 deletion, and how these genetic characteristics alter the transcriptional profile. Exome sequencing data revealed high-impact mutations in the DG region for five genes, which are already highly connected to DGS. Among these, when compared with hiPSC-EC expression data, we found that all family members carry the same in-del variants in CLTCL1 in homo/hemizygous form. An enrichment of other minor variations of M can also be observed, and the expression level in cells of DGS patients was consistently lower than that of the controls. CLTCL1, known for its role in clathrin-mediated endocytosis, is generally recognized to be expressed in endothelial cells due to its involvement in endocytosis and intracellular trafficking. However, specific studies on its expression in endothelial cells are limited; therefore, further data are needed to clarify its role in DGS. In endothelial cells, COMT plays a significant role in regulating vascular function and maintaining endothelial homeostasis. A high-impact variant in this gene also occurs in family members in homo/hemizygous form, except for GM (who is heterozygous), and its increased expression in hiPSC-EC of DGS samples can be observed. SCARF2 plays a crucial role in endothelial cell function, contributing to processes such as lipid metabolism, inflammation, and maintaining vascular integrity. High-impact SNPs and in-dels also occur in this gene for GF, F (who do not have vascular symptoms) and CH. RNA expression in hiPSC-EC was most affected in CH, suggesting that these variants may indeed influence endothelial cell formation and function. CLDN5, a tight junction component specific to endothelial cells, has been associated with heart development [30, 31]. We identified a hemizygous nonsense mutation in CLDN5 only in M, which may result in reduced expression in hiPSC-EC. TBX1, the most studied DGS-related gene, is not expressed in hiPSC-EC.

DGCR8 (DiGeorge Syndrome Critical Region 8) is the most frequently studied DGS-linked gene, along with TBX1. It is part of the microprocessor complex; disturbance of DGCR8 levels results in the alteration of microRNA biosynthesis and, thereby, in tissue developmental processes, cell proliferation, differentiation and cardiovascular development [32-34]. Dgcr8 deficiency leads to reduced cell proliferation and viability of vascular smooth muscle cells in mice [35]. To investigate the specific roles of DGCR8 in various cellular pathways, earlier, we established a hESC line carrying a monoallelic DGCR8 mutation by using the CRISPR-Cas9 system. We showed that DGCR8 heterozygous mutation results in only a modest effect on the mRNA level (by 20%) but a significant decrease at the protein level (40–60% of the control) [36]. We found lower DGCR8 mRNA expression levels in undifferentiated hiPSC from DGS patients than in the control group. However, although the levels varied during differentiation, they remained the lowest in samples of CH throughout all developmental time points, which may reflect a connection between DGCR8 and the severity of the developmental defect.

Exome sequencing was also used to analyze the number of high- and moderate-impact LOH variants occurring in the same genes. An increasing number of variations was associated with worsening symptoms in GF, M and CH. Of particular interest are variations in FAT1 in CH and VCAN in M, which are high-scoring genes in the MANTIS analysis for the development of cardiovascular symptoms. Out of these, FAT1 and VCAN showed abundant expressions in hiPSC-ECs. FAT1 (FAT Atypical Cadherin 1) is a member of the cadherin superfamily, a group of integral membrane proteins characterized by the presence of cadherin-type repeats, which function as adhesion molecules and thereby may play a role in developmental processes and cell communication. VCAN, a member of the aggrecan/versican proteoglycan family, is a major component of the extracellular matrix. As this protein is involved in cell adhesion, proliferation, migration, and angiogenesis, and plays a central role in tissue morphogenesis and maintenance, its high-impact variant in M would likely contribute to a cardiovascular phenotype. Based on WES and mRNA-seq data for symptomatic DGS family members, among the high/moderate-impact variations, CLDN5 and VCAN in M, while SCARF2 and FAT1 variations in CH may cause altered differentiation in endothelial cells.

STRING analysis shows an interesting correlation in the case of CH for TGFBR1-TGFB3-COL1A1, which also highlights the role of ECM in the in vitro cellular phenotype and the appearance of vascular symptoms. TGF-β is known to stimulate the expression of various extracellular matrix components, including collagens [37]. COL1A1 encodes the alpha-1 chain of type I collagen, the most abundant collagen in the human body, which plays a crucial role in providing structural support to tissues. CH carries a moderate impact, missense mutation in the TGFB1 (homozygous at position 41858876 C to G change) and TGFR1 (heterozygous at position 101911508 A to G change) genes, which may also contribute to the differential expression of collagens and thus the ECM composition.

Among the highly significant differentially expressed genes in DGS patients, GSTM1 (Glutathione S-transferase Mu 1) was the least expressed; its dysregulation may lead to vascular diseases and vascular remodelling partly via aberrant lipid peroxidation, oxidative stress, cellular damage, and inflammation. GSTM1 impacts various cellular processes, like cell proliferation and migration in vascular cells, thereby affecting the severity, manifestation, or exacerbation of cardiovascular anomalies in DGS [38]. However, differential expression showed that more genes were overexpressed in hiPSC-EC, which may indicate compensatory mechanisms in DGS.

We acknowledge the limitations of our study, including the small sample size and the absence of in vivo complexity. Despite these challenges, iPSC-based techniques offer distinct advantages for studying developmental disorders such as DGS. Our findings reveal significant differences between DGS patients and healthy controls on average. Moreover, individual analysis shows a progressive worsening of symptoms at cellular and molecular levels across generations. The further systematic analysis of our data and study of the generated cell lines provides a foundation for exploring additional DGS symptoms, such as immunological, neural, or calcium homeostasis defects.

In summary, we have demonstrated here that disease-specific cell types differentiated from hiPSCs exhibit phenotypic alterations and can be utilized to reveal genotype-phenotype correlations and disease mechanisms, thereby serving as a personalized medicine approach. For deep clinical phenotyping of each participant, we used a unified list of clinical signs and their corresponding Human Phenotype Ontology terms and numbers. Gene-to-phenotype algorithms, validated using large registries, confirmed our in vitro findings. The development of biomarkers to segment diseases into better understood, molecularly characterized subtypes depends on access to well-defined patient populations and patient biological samples along with longitudinal clinical data and medical and treatment histories. This project provides novel preclinical data needed to understand and exploit this previously unrecognized window in stratification for DGS with cardiovascular manifestations. Modulating and testing committed cardiomyocytes and endothelial cells differentiated from stem cells may be a viable target screening route that could be developed into a strategy in human cells. Our study also provides new insights into the pathogenesis and development of the cardiovascular aspects of DGS.

## 4. METHODS

### 4.1. Patient screening.

Over the last 18 years, 100 children with congenital cardiovascular defects and suspected DGS were referred to the Cytogenetic Laboratory of the 2nd Dept of Paediatrics, Semmelweis University [39]. The cytogenetic diagnosis was carried out in most cases by FISH (N25, Tuple, for TBX1) and complemented by microarray-based analysis in the case of complex rearrangements. DGS was identified in 60 patients. From this cohort, we identified a family spanning three generations affected by DGS, with a noted exacerbation of symptoms across generations. At the time of blood sampling, the ages of the family members were as follows: grandfather, 55–60 years; grandmother, 50–55 years; father, 30–35 years; mother, 25–30 years; and child, 0–5 years. Patient characterization is described in the Results section.

### 4.2. Generation of human induced pluripotent stem cells.

According to the permission for reprogramming and the study by the Human Reproduction Committee of the Hungarian Health Science Council (ETT HRB- Approval number: 42592-2/2016-EHR), blood samples were obtained from patients and healthy relatives after written informed consent. Peripheral blood mononuclear cells (PBMC) were isolated using cell separation tubes containing sodium citrate (BD Vacutainer) as per the manufacturer's instructions. 5x10^5^ cells/well were plated in 24-well plates in 1 ml PBMC medium consisting of StemPro-34 medium supplemented with StemPro-34 Supplement, completed with 2 mM GlutaMax, 10 μM β-mercaptoethanol, 1% Antibiotic-Antimycotic and cytokines: 20 ng/ml IL-3, 20 ng/ml IL-6, 100 ng/ml FLT-3, 100 ng/ml SCF. Half of the medium was replaced daily with fresh PBMC medium for 2 days. Reprogramming of PBMCs was performed by Sendai virus (SeV) vectors (CytoTune-iPS 2.0 Sendai Reprogramming Kit, Invitrogen) expressing four transcription factors – Oct3/4, Sox2, Klf4 and c-Myc in the cells. The reprogramming factors were removed the next day, and the cells were replated in 1 ml fresh PBMC medium/well on a 24-well plate. After 24 hours, enlarged/swollen cells were observed. The next day, the cells were seeded on mitomycin C-treated mouse embryonic fibroblast cell culture (CF-1 MEF feeder cells, Applied StemCell) in 2 ml supplemented StemPro-34 medium/well on a 6-well plate (without cytokines). Half of the medium was changed daily for 2 days. Transitioning into hiPSC medium occurred by replacing half of the medium with hiPSC medium. hiPSC medium consisted of KO-DMEM/F-12 supplemented with KO-Serum Replacement, 100 mM MEM Non-Essential Amino Acids, 2 mM GlutaMax, 10 mM β-mercaptoethanol, 4 ng/ml bFGF and 1% Antibiotic-Antimycotic. From the next day, the medium was replaced with fresh hiPSC medium daily. When hiPSC colonies were ready for transfer, they were picked using a sterile needle and pipette and were transferred onto MEF culture in hiPSC medium for expansion. Later, colonies were picked and transferred to Matrigel-coated plates mTeSR1 medium for expansion and culturing in a feeder-free monolayer. Cells were passaged with Accutase when they reached ~ 90% confluency and plated in mTeSR1 medium containing 10 mM Rock inhibitor. The medium was changed the next day to mTeSR1. A total of six hiPSC lines were stabilized, characterized and used in this work, which were registered on the Human pluripotent stem cell registry (https://hpscreg.eu/) website, and can be found under the following registered names: GM (grandmother) - RCNSi003-A, GF (grandfather) -RCNSi005-A, F (father) - RCNSi006-A, M (mother) - RCNSi007-A, CH (child) - RCNSi008-A and RCNSi008-B.

### 4.3. Fluorescence-activated cell sorting (FACS).

Cells were dissociated with Accutase and washed with PBS containing 0.5% BSA. Cells were incubated with SSEA4-APC (IgG3, R&D System) or IgG3-APC isotype control antibodies in PBS + 0.5% BSA in a 37°C water bath in a shaker for 30 minutes. Cells were washed with PBS + 0.5% BSA, and 7-AAD (Abcam) was added to mark non-viable cells. BD FACSCalibur Cell Analyser was used to detect SSEA4 positivity.

### 4.4. Spontaneous differentiation of hiPSC.

To evaluate pluripotency, spontaneous differentiation was performed as described previously [40].

### 4.5. Karyotype analysis.

After 15 passages, mycoplasma-free culture conditions and the karyotype were tested in the Laboratory of Genetics, 2nd Department of Pediatrics, Semmelweis University.

### 4.6. Multiplex ligation-dependent probe amplification.

Given that the FISH method does not have sufficient sensitivity to detect small deletions or duplications (< 40 kb) within 22q11.2, we used multiplex ligation-dependent probe amplification (MLPA) for the whole region [41]. DNA was isolated from hiPSC using Wizard Genomic DNA Purification Kit according to the manufacturer's instructions. MLPA was performed using MLPA probes designed for 22q11.2 deletion analysis (MRC Holland SALSA MLPA Probemix P250-B2 DG) following the manufacturer's instructions.

### 4.7. STR analysis.

Analyses were performed by UD-GENOMED Medical Genomic Technologies Ltd. (Hungary) using GenePrint^®^ 10 System (Promega).

### 4.8. Whole exome sequencing and selection of candidate gene variables.

Genomic DNA from hiPSC lines was isolated using NucleoSpin DNA purification kit (MACHEREY-NAGEL) according to the manufacturer's instructions. Whole exome sequencing was performed by BGI with 100x sequencing depth. Data analysis occurred using IGV (Integrative Genomics Viewer) software and custom bash scripts. We also searched for de novo SNP and indel variants in the mother and child. SnpEff performed genetic variant annotation and functional effect prediction [42].

### 4.9. Cardiomyocyte differentiation.

A previously described protocol was applied and optimized for cardiomyocyte differentiation [11]. hiPSC were cultured in a monolayer on Matrigel-coated 12-well plates in mTeSR medium. At ~ 80% confluency, the medium was changed to RPMI 1640 medium completed with B27 (without insulin), containing 8 mM CHIR99021. Medium was changed two days later to RPMI 1640 medium completed with B27 (without insulin). The next day, medium was changed to RPMI 1640 medium B27 (without insulin) containing 2.5 mM C59. Medium was changed two days later to RPMI 1640 medium completed with B27 (containing insulin), and cells were kept in this medium for further maintenance, changing every second day. Passaging cardiomyocytes occurred on day 25 of cardiac differentiation using TrypLE Express, and cells were plated in RPMI 1640 medium completed with B27 (containing insulin) supplemented with 10% FBS and 10 mM Rock inhibitor. 200.000 cells/well were seeded in 24-well format for immunostaining and calcium transient measurements. Medium was changed the next day to RPMI 1640 medium completed with B27 (containing insulin).

### 4.10. Analysis of calcium transients.

For functional assessment of cardiomyocytes, hiPSC-CMs were passaged on day 25 (2×10^5^ cells per well) and cultured as a confluent monolayer in Matrigel-coated 24-well plates. The recording was performed 10 days post-plating. Cells were loaded with 1 μM Fluo-4 AM in CM culturing medium for 30 minutes. The medium was changed to a recording medium consisting of DMEM without phenol red containing 1 mM sodium pyruvate, 2 mM GlutaMax, 25 mM glucose, and 10 mM HEPES, with pH 7.4. The cells were further incubated for 5 minutes prior to imaging. Calcium transients were recorded using ImageXpress high-content screening microscope (Molecular Devices) using a 488 nm filter. Video analysis was performed using Image J software. Specific parameters calculated were frequency, time to transient peak (Tp) and 90% decay (T90). Measurements were performed with at least three independent biological repetitions.

### 4.11. Endothelial differentiation protocol.

The differentiation protocol described in detail by our group [43] was optimized for the iPSC lines. Briefly: human PSCs were detached using Versene solution and collected by centrifugation at 1200 rpm for 5 min. The supernatant was removed by aspiration, and the cell pellet was resuspended in mTeSR1 media. 10^5^ cells were seeded onto 24-well Matrigel-coated (1:50) plate in mTeSR1 medium containing Y-27632 (10ng/ml, Gibco, UK) and grown for two days (day – 2) at 37°C with 5% CO_2_. At day 0, the media was replaced with mTeSR1 containing the growth factors Activin A (R&D systems, UK, #338-AC), bone morphogenic protein 4 (BMP4, R&D systems, UK, #314-PB/CF), basic fibroblast growth factor (bFGF, R&D systems, UK, #4114-TC) and VEGF-A (Peprotech, UK, #100 – 20). All factors were used at a concentration of 10 ng/ml. After 24 hours (day 1), the mTeSR1 medium supplemented with the four factors was replaced with Stemline II hematopoietic stem cell medium (herein Stemline II, Sigma-Aldrich, UK) containing 3 factors: BMP4, bFGF and VEGF-A (all 10ng/ml). Samples for RNA profiling were collected on days 0, 5, 12 and 19 of the differentiation protocol. On day 12, CD31-positive hPSC-ECs were selected by fluorescence-activated cell sorting (FACS), using a FACSAria Cell Sorter (BD Biosciences, NJ, US). Briefly, cells were washed once in PBS, incubated with 0.05% trypsin-EDTA (TE) solution at 37 °C for 8 mins, and detached mechanically by pipetting. The TE solution was then neutralized in Stemline II media supplemented with 10% foetal bovine serum (FBS, Gibco, UK), and cells were collected by centrifugation at 1200 rpm for 5 mins. The cell pellet was resuspended in PBS w/o Ca^2+^-Mg^2+^ supplemented with 1% FBS (FACS blocking buffer). Cells were then incubated with anti-human CD31 AlexaFluor 488-conjugated (1:20; BD, UK, #557703) diluted in FACS clocking buffer for 25 mins at 4°C for cell surface double labelling. Sorted cells were collected in collection media (40% Stemline II medium, 40% endothelial growth factor medium-2 (EGM2, Lonza, UK), 20% FBS and 1% penicillin/streptomycin solution (P/S, Gibco, UK). After sorting, cells (2500/cm^2^) were seeded onto type IV collagen-coated (Sigma-Aldrich, UK, #C7521) TCP plates, with replating media (50% EGM2 + 50% Stemline II supplemented with BMP4, bFGF and VEGF-A (all 10 ng/ml) and 1% P/S. After 24 hours, the medium was replaced with 75% EGM2, 25% Stemline II supplemented with BMP4, bFGF and VEGF-A (all 10 ng/ml) and 1% P/S. Sorted cells (day 12 hPSC-ECs, passage 0) were fed every other day as follows: day 14, with media containing 75% EGM2, 25% Stemline II supplemented with BMP4, bFGF and VEGF-A (all 10 ng/ml); day 16 and later, with 100% EGM2 media, until they reached 80% confluency. After reaching 80% confluency (after day 19), hPSC-ECs were subcultured (passage 1) at 10,000–12,000 cells/cm^2^ in fully supplemented EGM2 (standard EGM2) and detached using 0.05% TE. EGM2 medium was replaced every other day until the cells reached 80% confluency.

### 4.12. Immunostaining of hiPSC and differentiated derivatives.

hiPSC and differentiated cells were fixed with 4% paraformaldehyde and permeabilized with a blocking buffer consisting of PBS, 2 mg/ml BSA, 0.1% Triton X-100 + 5% donkey serum. Cells were stained using primary antibodies (Oct3/4, Nanog, B-III-tubulin, Nestin, SMA, BMP4, AFP, SOX 17, cardiac troponin I (TNNI), CD31 and with appropriate secondary antibodies where necessary (Table S4), and nuclei were labelled with DAPI. HiPSC-CMs and hPSC-ECs were assessed using the ImageXpress Micro XLS high content screening device (Molecular Devices). Cell morphology of hiPSC-CM has been evaluated for sarcomere reorganization (entropy as an endpoint), cell size, and cell number (reflecting cell death and proliferation balance). Six fields of view (approximately half of the total well surface area) were imaged using a DAPI filter cube (ex. 377/50 nm, em. 447/60 nm), a FITC filter cube (ex. 482/35 nm, em. 536/40 nm) with a 10x Nikon objective (Plan Fluor, NA = 0.3).

### 4.13. mRNA quantification by qRT-PCR of hiPSC and hiPSC-CM.

hiPSC were lysed in TRIzol, and total RNA was isolated using Direct-zol RNA MiniPrep Plus kit according to the manufacturer's instructions. cDNA synthesis was carried out using Promega Reverse Transcription System according to the manufacturer's instructions. qRT-PCR was performed using TaqMan Gene Expression Assays (see the assays in Table S4), and expression levels were measured using QuantStudio 5 Real-Time PCR System. mRNA levels were quantified based on the 2^−ΔCt^ method, using GAPDH as endogenous control. Measurements were performed with at least three independent biological repetitions.

### 4.14. RNAseq analysis.

RNA integrity and quantitation were assessed using the RNA Nano 6000 Assay Kit of the Bioanalyzer 2100 system (Agilent Technologies, CA, USA). The Ribo-Zero Plus rRNA Depletion Kit was used to remove abundant RNA using enzymatic depletion (Illumina, CA, USA). Sequencing-ready libraries were prepared with Nextera XT DNA Library Preparation Kit (Illumina, CA, USA) following the manufacturer's recommendations. Products were purified with AMPure XP beads (Beckman Coulter, CA, USA) and library quality was assessed on the Agilent Bioanalyzer 2100 system (Agilent Technologies, CA, USA). According to the manufacturer's instructions, the clustering of the indexed samples was performed on a cBot Cluster Generation System using PE Cluster Kit cBot-HS (Illumina, CA, USA). After cluster generation, the libraries were sequenced on an Illumina HiSeq 2000 platform, and 100bp paired-end reads were generated. Raw reads in FASTQ format were first processed through fastqc. Read mapping against a recent ENCODE human release was performed using the Spliced Transcripts Alignment to a Reference (STAR) software. FeatureCounts was used to count the read numbers mapped for each gene. And then, the RPKM (Reads Per Kilobase of exon model per Million mapped reads) of each gene was calculated based on the length of the gene and the reads count mapped to this gene. RPKM considers the effect of sequencing depth and gene length on the read count at the same time.

Hierarchical cluster analysis was performed on RNAseq-based transcriptomics profiling of differentiating hiPSC-derived endothelial cells from patients with DiGeorge syndrome and healthy relatives. Enrichment analysis was carried out using the Ingenuity Pathway Analysis platform (Qiagen). The 15 GO processes with the largest gene ratios are plotted in order of gene ratio. Differential expression analysis between two conditions/groups (three biological replicates per condition) was performed using the DeSeq2 R package. DeSeq2 provides statistical routines for determining differential expression in gene expression data using a model based on the negative binomial distribution. Principal component analysis for differentiating (days 0 and 5), as well as differentiated hiPSC-EC (day19) samples in the six cell lines, was performed by GraphPad Prism 10.0 and the matplotlib python package. Functional enrichment analysis of protein-protein interaction networks was performed by String DB (string-db.org). RNAseq datasets are deposited in NCBI Sequence Read Archive (SRA, BioProject accession number No. PRJNA1196389), database, https://www.ncbi.nlm.nih.gov/sra/PRJNA1196389.

### 4.15. Statistical analysis.

Data were plotted in GraphPad Prism Version 9.4 and expressed as mean ± SEM. Unpaired t-tests were used to compare the control and DGS groups. Comparisons between multiple datasets were analyzed using one-way ANOVA. Differences at the level of p < 0.05 were considered statistically significant, and the following labelling key was used: * p < 0.05, ** p < 0.01, *** p < 0.001, **** p < 0.0001.

## Figures and Tables

**Table 1.** Clinical features of family members affected by 22q11.2 deletion and healthy relatives. Clinical symptoms of patients show increasing severity when the disease is inherited. Anomalies of the cardiovascular system, thymus, parathyroid gland, renal system and facio-skeletal system were revealed during clinical evaluation of the infant and mother. At the same time, the grandfather showed only mild symptoms affecting the facial-skeletal and neuropsychological systems. Right aortic arch (RAA) with an aberrant left subclavian artery (LSCA). Blue “−“ depicts, no clinical presentation, red “+”, clinical presentation.

	GM	F	GF	M	CH
Cardiovascular system				
Pulmonary atresia	−	−	−	−	+
Ventricular septal defect	−	−	−	−	+
Atrial septal defect type ii	−	−	−	−	+
RAA+ LSCA	−	−	−	−	+
Stenosis of aortopulmonary collateral arteries	−	−	−	−	+
Vascular ring	−	−	−	+	+
Third-deyee atrioventricular block	−	−	−	−	+
Neuropsychological system					
Dysarthria	−	−	+	−	N/A
Hypotonia	−	−	−	−	+
Psychiatry symptoms	−	−	+	−	−
Language development delay	−	−	+	−	N/A
Neurodevelopmental delay	−	−	−	−	+
Craniofacial-skeletal system					
facial anomaly	−	−	+	+	+
Micrognathia	−	−	−	−	+
long face	−	−	+	+	−
low-set ears	−	−	−	+	+
hooded eyelids	−	−	+	+	+
tubular nose	−	−	+	+	−
alar hypoplasia	−	−	−	−	−
velopharyngeal insufficiency	−	−	−	−	−
Thymus and parathyroid gland					
T-cell deficiency	−	−	−	+	+
recurrent infections	−	−	−	+	+
Thymic hypoplasia	−	−	−	+	+
Immunodeficiency by laboratory analysis	−	−	−	+	+
Hypocalcaemia to hypoparathyroidism	−	−	−	+	+
Renal system					
Pyslectasis	−	−	−	−	+
Medullary sponge kidney	−	−	−	+	−
Hypercalciuria	−	−	−	+	−
Other					
Anaemia	−	−	−	+	+
Hyperthyroidism	−	−	+	−	−
Inguinal hernia	−	−	+	−	−
